# Computation screening and molecular docking of FDA approved viral protease inhibitors as a potential drug against COVID-19 

**Published:** 2020

**Authors:** Abdorrahim Absalan, Delaram Doroud, Mostafa Salehi-Vaziri, Hooman Kaghazian, Nayebali Ahmadi, Fatemeh Zali, Mohamamd Hassan Pouriavali's, Seyed Dawood Mousavi-Nasab

**Affiliations:** 1 *Department of Medical Laboratory Sciences, Khomein University of Medical Sciences, Markazi Province, Iran*; 2 *Department of Research and Development, Production and Research Complex, Pasteur Institute, Tehran, Iran*; 3 *Viral vaccine research center, Pasteur institute of Iran*; 4 *Department of Arboviruses and Viral Hemorrhagic Fevers, Pasteur Institute of Iran*; 5 *Proteomics Research Center, Department of Medical Lab Technology, Faculty of Paramedical Sciences,Shahid Beheshti University of Medical Sciences, Tehran, Iran*; 6 *Department of Clinical Biochemistry, Faculty of Medicine, Tehran University of Medical Science, Tehran, Iran *

**Keywords:** COVID-19, Main protease, Mpro/6LU7, Molecular docking, Viral antiprotease drugs

## Abstract

**Aim:**

This study demonstrated potent inhibitors against COVID-19 using the molecular docking approach of FDA approved viral antiprotease drugs.

**Background:**

COVID-19 has now spread throughout world. There is a serious need to find potential therapeutic agents. The 3C-like protease (Mpro/6LU7) is an attractive molecular target for rational anti-CoV drugs

**Methods:**

The tertiary structure of COVID-19 Mpro was obtained from a protein data bank repository, and molecular docking screening was performed by Molegro Virtual Docker, ver. 6, with a grid resolution of 0.30 Å. Docking scores (DOS) are representative of calculated ligand-receptor (protein) interaction energy; therefore, more negative scores mean better binding tendency. Another docking study was then applied on each of the selected drugs with the best ligands separately and using a more accurate RMSD algorithm.

**Results:**

The docking of COVID-19 major protease (6LU7) with 17 selected drugs resulted in four FDA approved viral antiprotease drugs (Temoporfin, Simeprevir, Cobicistat, Ritonavir) showing the best docking scores. Among these 4 compounds, Temoporfin exhibited the best DOS (-202.88) and the best screened ligand with COVID-19 Mpro, followed by Simeprevir (-201.66), Cobicistat (-187.75), and Ritonavir (-186.66). As the best screened ligand, Temoporfin could target the Mpro with 20 different conformations, while Simeprevir, Cobicistat, and Ritonavir make 14, 10, and 10 potential conformations at the binding site, respectively.

**Conclusion:**

The findings showed that the four selected FDA approved drugs can be potent inhibitors against COVID-19; among them, Temoporfin may be more potent for the treatment of the disease. Based on the findings, it is recommended that in-vitro and in-vivo evaluations be conducted to determine the effectiveness of these drugs against COVID-19.

## Introduction

 Recently, a severe, highly contagious viral disease of the coronavirus family originated in the Hubei province of China and quickly spread throughout the world. The causative agent was a novel coronavirus termed Coronavirus disease 2019 (COVID-19) (1, 2). Symptoms associated with this new coronavirus include fever, cough, myalgia or fatigue, and pneumonia, but the symptoms can be anything (3, 4). Coronaviruses infect human beings and a wide range of animals, causing severe acute respiratory syndrome CoV (SARS-CoV) which, about a decade ago, evolved into the Middle East respiratory syndrome coronavirus (MERS-CoV) that was diagnosed in Saudi Arabia (5-7). They are enveloped viruses with a positive-single stranded, large RNA genome .The SARS genome is comprised of ~30,000 nucleotides and encodes 2 overlapping polyproteins required for viral replication and transcription, namely ppla and pplb with molecular weights of 450 and 750 kDa, respectively (8, 9). Polyproteins are cleaved to different functional proteins of spike surface glycoprotein, matrix protein, small envelope protein, nucleoprotein, replicase, and polymerase. This process is performed predominantly by a 33.8 kDa main protease (Mpro), and because of its similarity to protease picornavirus 3C, it is also referred to as the 3C-like protease (1, 10, 11). For several reasons including an essential role in the viral life cycle, a lack of human homologues, and the substantial homology between human and animal coronavirus, Mpro has been identified as an attractive target for antiviral drugs (12). The function of a protein is dependent upon its tertiary (3D) structure, and clarification of the 3D structure helps immunoinformatics and chemoinformatics and can result in the design of novel vaccines and inhibitory medicines (13). The crystal structure of the COVID-19 Mpro (PDB ID: 6lu7) was recently presented, providing researchers with an opportunity to design wide-spectrum anti-CoV inhibitors (14). The drug 5, 10, 15, 20‐Tetra(m‐hydroxyphenyl) chlorin (Mthpc) with the generic name Temoporfin was introduced in 2000 and has been the subject of investigations for almost two decades (15). Temoporfin, a photosensitizer drug, was approved by the European Union for the treatment of squamous cell carcinoma of the head and neck (16). Some studies have shown that as a broad spectrum drug, Temoporfin not only has anti-flaviviral activities, but also protects animals from a lethal challenge by ZIKV (17). In vitro methods such as developing a culture system are time-consuming and expensive in comparison with in silico prediction methods. Molecular docking is a way to predict probable interactions between ligand-protein complexes and a useful method for finding the best therapeutic candidates from an existing library (18). At present, there are no vaccines or effective antiviral therapies for COVID-19. The current study aimed to identify potential drugs against COVID-19 among FDA approved viral protease inhibitors using the computation molecular docking approach. The results could accelerate the discovery of antiviral compounds with clinical potential in the combat against COVID-19 infection. 

## Methods

**Figure 1:**
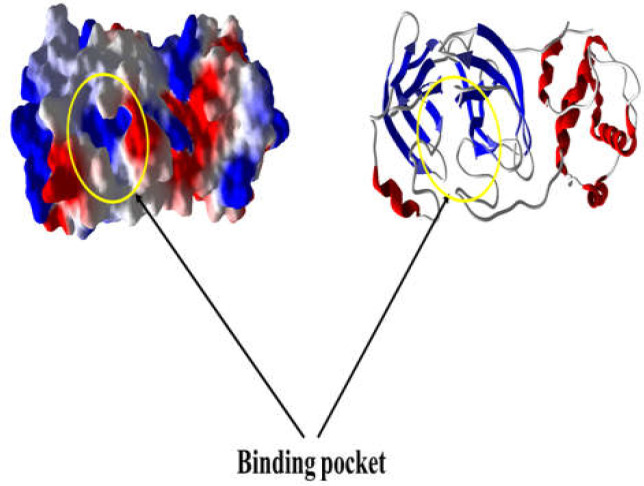
The 3D-structure of COVID-19 Mpro/6LU7 that shows the binding pocket. The docking studies of 16 AFD-approved drugs were evaluated in this binding site

This docking study evaluated the interaction between virus protease and selected approved drugs. The three-dimensional structures (3D) of chemicals were downloaded from ZINCDocking repository in Schordinger's format (SDF) (19). The crystallographic structure of the COVID-19 main protease was recently made publicly available through the protein data bank (PDB) repository [PDB code 6LU7]. This structure is a synthetic construct expressed in Escherichia coli BL21 (DE3) and structurally determined by X-ray diffraction with a resolution of 2.16 Å (20). During the crystallography process, the authors used an inhibitor with the chemical name of “N-[(5-METHYLISOXAZOL-3-YL) CARBONYL] ALANYL-L-VALYL-N~1~-((1R, 2Z)-4-(BENZYLOXY)-4-OXO-1-BUT-2-ENYL)-L-LEUCINAMIDE” (leucinamide). Because leucinamide is used as an effective inhibitor, its position in the protein was considered as the main binding pocket, and the docking process focused mainly on this pocket (Figure 1). 

Molegro Virtual Docker (MVD), ver. 6, was used for this molecular docking study. The docking was done in these steps, and with these statuses and considerations: importing SDF and protein 3D-structures (without water molecules and associated ligand) into the docking environment; searching for all probable cavities on the protein surface; defining the binding site and grid space (with 0.3 Å); setting the search algorithm on the energy-minimization and optimization of hydrogen bonds; running the software and saving the docking results for subsequent analysis. Note that the docking scores (DOS) are representative of calculated ligand-receptor (protein) interaction energy; therefore, more negative scores mean better binding tendency. Initially, a screening approach was used to select 17 ligands. Then, another docking was done using each of the four best ligands separately, a more accurate algorithm (RMSD calculation by automorphism), and an energy penalty of 100. The main binding pocket of interest was the inhibition site of the leucinamide molecule, as mentioned before. To better interpret the results and considering a mean evaluation of ligand-protein docking, the MolDock scores were exported and defined in SPSS ver. 20 software. Statistical analysis on DOS was done using one-way analysis of variances (ANOVA) with a 95% confidence interval. 

## Results

**Figure 2:**
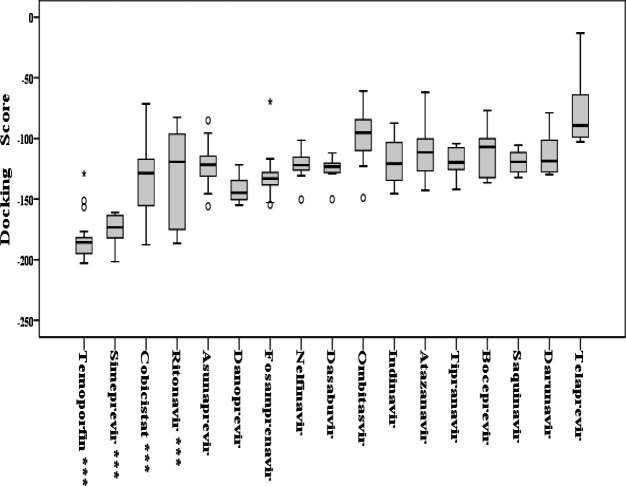
Comparative box-plots for means of MolDock scores of ligand-protein interactions; selected FDA approved viral antiprotease drugs were docked with the structure of COVID-19 Mpro/6LU7. Four drugs with the highest scores are marked with three asterisks

**Figure 3:**
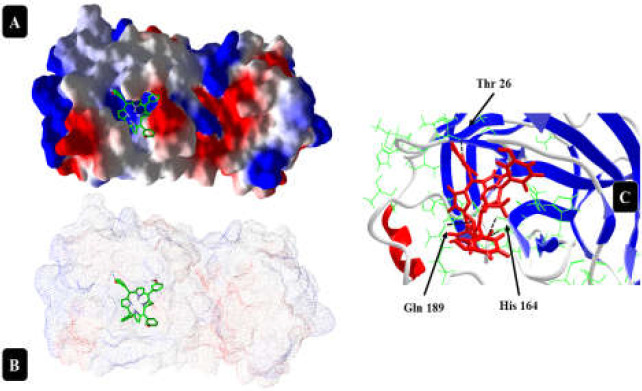
Temoporfin interaction with 6LU7 protein at the best position with the highest DOS (A and B); DOS= -202.883. As can be seen, Temoporfin makes four hydrogen bonds at this site with Thr 26, Gln 189, and His 164 (bifurcated) (C).

In the present study, molecular docking of 17 selected FDA approved viral antiprotease compounds was performed against COVID-19. Table 1 depicts detailed information of the order of the best docking scores, hydrogen bond energy, the chemical forms of each drug, and the FDA-approved application. Figure 2 shows the comparative box-plot for the mean of MolDock scores for the ligand-protein interactions of the selected FDA approved drugs. To save space and prevent the presentation of lesser important data, the docking results of Temoporfin and three other FDA approved viral antiprotease drugs (Simeprevir (Olysio), Cobicistat, and Ritonavir) that showed the best docking scores are presented. Figure 3 (A to C) depicts the interaction between Temoporfin and 6LU7 protein at the best position with the highest DOS. 

**Table 1:** Molecular docking analysis of viral protease inhibitors against COVID-19 major protease (6LU7). Candidate drugs sorted by the best docking scores

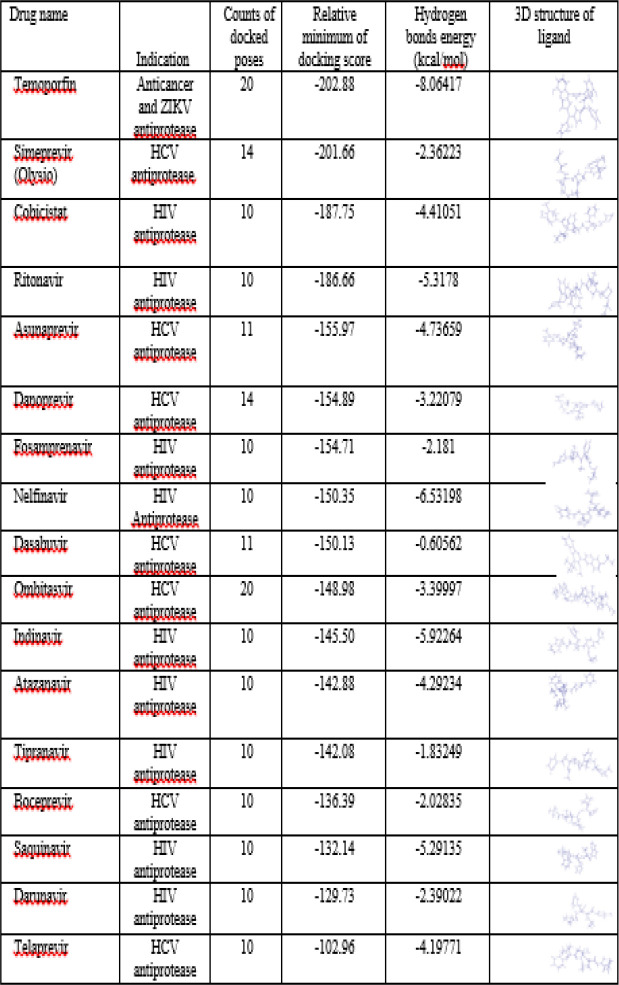

**Figure 4:**
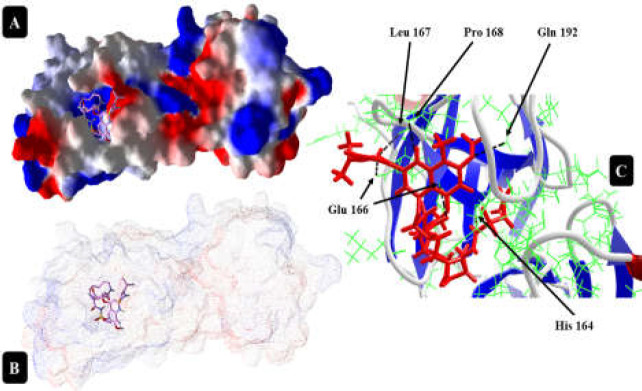
Simeprevir interaction with 6LU7 protein at the best position with the highest DOS (A and B); DOS= -201.663. As can be seen, Simeprevir makes six hydrogen bonds at this site with Glu 166 and Leu 167 (bifurcated), Glu 165, His 164, Pro 168, and Gln 192 (C)

**Figure 5:**
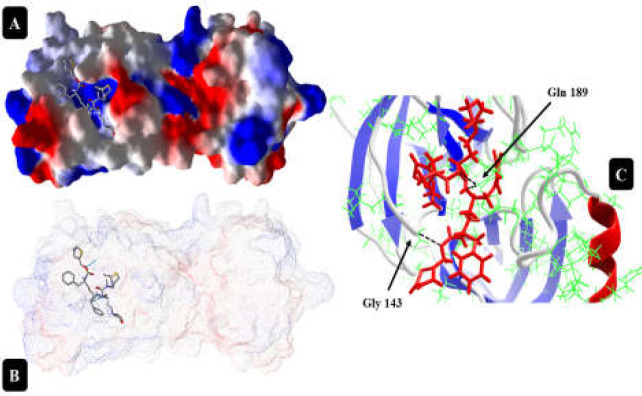
Cobicistat interaction with 6LU7 protein at the best position with the highest DOS (A and B); DOS= -187.749. At this site, Cobicistat makes three hydrogen bonds, two with Gln 189 (from atoms N11 and N23 of drug structure) and one with Gly 143 (C).

**Figure 6:**
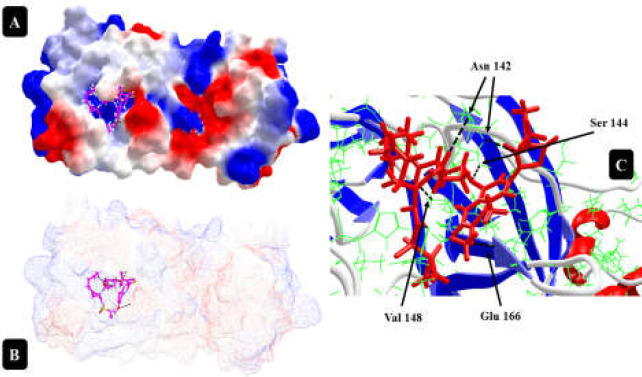
Ritonavir interaction with 6LU7 protein at the best position with the highest DOS (A and B); DOS= -186.66. At this site, Ritonavir (Norvir) makes five hydrogen bonds, two with Asn 142 (from atoms N41 and O12 of drug structure), one with Glu 166, Val 148 and Ser 144 (C).

Temoporfin exhibited the best DOS (-202.88) and the best screened ligand with COVID-19 Mpro/6LU7, followed by Simeprevir (-201.66), Cobicistat (-187.75), and Ritonavir (-186.66), successively. The present molecular docking showed that Temoporfin makes four hydrogen bonds with Thr 26, Gln 189, His 164 (bifurcated) in the binding pocket of Mpro/6LU7, and might be a potent treatment against COVID-19. Figure 4 (A to C) shows that Simeprevir docks with 6LU7 and makes six hydrogen bonds with Glu 166 and Leu 167 (bifurcated), Glu 165, His 164, Pro 168, and Gln 192. The molecular docking of HIV-1 antiprotease drugs with COVID-19 Mpro/6LU7 protein showed that Cobicistat makes three hydrogen bonds, two with Gln 189 and one with Gly 143 (Figure 5; A-C), and Ritonavir makes five hydrogen bonds, two with Asn 14, and one each with Glu 166, Val 148, and Ser 144 (Figure 6; A-C).

## Discussion

COVID-19 is a public health emergency for all countries, and efforts to create an efficient vaccine or drug for the prevention or treatment of this infection are essential. The approval of new compounds as drugs requires a significant number of years of investigations and a high cost. Therefore, repurposing already-approved drugs can be the best starting point for developing new therapies for COVID-19. Among coronavirus proteins, Mpro (6LU7) has multiple functions, including protease activity. Moreover, molecular docking exhibits the chance of interaction between ligand and protein which is dependent upon the affinity of docking, which is dependent upon the binding energy during the attachment process. To predict the effects of Temoporfin on COVID-19, this drug was compared with antiprotease drugs utilized against hepatitis C and HIV. The HCV NS3/4A protease is a trypsin-like serine protease and essential for the viral RNA replication complex. Simeprevir (Olysio) is a second generation antiviral drug indicated for the treatment of chronic hepatitis C infection (21, 22). Because of the high value of DOS in our virtual study and the minimal side effects for HCV patients, Simeprevir can be a valuable therapeutic option in treating COVID-19.

Remarkably, Cobicistat and Ritonavir, as two HIV-protease inhibitors, showed docking interaction results with lower free energy (-4.41051, -5.3178 respectively) than Simeprevir, while Simeprevir, because of its relative minimum docking score, was better than the other two. The findings demonstrated that Cobicistat and Ritonavir can be used as potential drugs against SARS-CoV2, similar to previous reports on the usefulness of anti-HIV-1 protease inhibitors for the treatment of SARS-CoV2 infection (23, 24).

The current study observed that Temoporfin is most potent and can result in a rapid resolution of COVID-19. Currently Temoporfin is applied in a single dose of 0.15 mg/kg body weight maintained for at least 5 days, enabling it to inhibit flaviviruses, including ZIKV and anal intraepithelial neoplasia related to HPV (25, 26). Scientific studies have demonstrated that Temoporfin is able to inhibit ZIKV viremia and protect 83% of the viremia in a mouse model (17, 27). There is evidence that Temoporfin activates macrophages, increases phagocytosis, and results in tumor necrosis factor-α (TNF-α) and nitric oxide (NO). One study reported that Temoporfin is able to inhibit ZIKV protease activity by interacting with NS2B – NS3 protease. Also notable, this drug reportedly inhibits viral replication by inhibiting the interactions itself and without photoactivation (25, 28). Data from the current study showed that Temoporfin, as the best screened ligand, could target the Mpro/6LU7 with 20 different conformations, while Simeprevir, Cobicistat, and Ritonavir made 14, 10, and 10 potential conformations at the binding site, respectively. A notable result from the hydrogen bond is the presence of common amino acid residue in the binding pocket of the drugs. His 164 and Gln 189 were common residues among the four mentioned drug-protein interactions (Figures 3 to 5). Therefore, it is possible that at least two residues could be hot points for targeting the COVID-19 Mpro/6LU7. Most attempts to develop coronavirus protease inhibitors have met with limited success, possibly due to the presence of a flexible occluding loop preventing the putative inhibitors to access the binding pocket (29); however, in vitro and in vivo experiments are needed.

The results showed that four FDA approved viral antiprotease drugs, namely Temoporfin, Simeprevir, Cobicistat, and Ritonavir can be potent inhibitors of Mpro/6LU7 of SARS CoV-2. Among the known inhibitors, Temoporfin had a better chance of binding and inhibiting Mpro/6LU7 than HCV and HIV-1 protease inhibitors, and may be prescribed alone or in combination with other drugs. Another important finding of the current docking study was that the residues His 164 and Gln 189 may be critical amino acids for targeting COVID-19 Mpro/6LU7. Based on the results, in vitro and in vivo evaluations to determine the effectiveness of these drugs against COVID-19 are recommended.
